# Supplementary material for: Epidemiologic Analysis of Taiwanese Patients with Idiopathic Pulmonary Fibrosis
Source: Healthcare (Basel). 2020 Dec 21;8(4):580. doi: 10.3390/healthcare8040580 (PMC7767390; doi:10.3390/healthcare8040580)
Supplement: Supplementary file 1 [file healthcare-08-00580-s001.zip › supplementary Table S2.docx]

Table S2. NHI codes for Procedure of Daignosing Lung Disease

| NHI code  of medical order | Description |
| --- | --- |
| 15020 B | Imprint bronchial biopsy |
| 19007 B | Ultrasonic guidance for needle placement (eg, biopsy, aspiration, injection) |
| 25009 B | Muscle biopsy |
| 28009 B | Thoracoscopy with biopsy |
| 28026 B | Mediastinoscopy with biopsy |
| 28030 C | Endoscopic biopsy |
| 33103 B | Computed tomography (C. T.) guide biopsy |
| 51027 B | Excision biopsy-normal |
| 51028 B | Excision biopsy-special |
| 56015 B | Pleural biopsy |
| 92021 B | Biopsy, soft tissue |
| 92022 B | Biopsy, hard tissue |
| 92067 B | Biopsy for precancerous lesion, soft tissue |
| 92068 B | Biopsy for precancerous lesion, hard and soft tissue |
| 67051 B | Thoracoscopic wedge or partial resection of the Lung |
| 25004 C | Surgical pathology, level IV |
| 28006 C | Bronchoscopy |
| 33070B | Computed tomography (C. T.) - without contrast |
| 33071B | Computed tomography (C. T.)- with contrast |
| 33072B | Computed tomography ( C. T.) -with/without contrast |
| 33090B | Low osmolarity or nonionic contrast |

Definition of abbreviation: NHI, National Health Insurance
