# Supplementary material for: Epidemiologic Analysis of Taiwanese Patients with Idiopathic Pulmonary Fibrosis
Source: Healthcare (Basel). 2020 Dec 21;8(4):580. doi: 10.3390/healthcare8040580 (PMC7767390; doi:10.3390/healthcare8040580)
Supplement: Supplementary file 1 [file healthcare-08-00580-s001.zip › Supplmentary Table S1.docx.doc]

Table S1. ICD-9-CM Codes for Interstitial Lung Disease Other Than Idiopathic Pulmonary Fibrosis

| ICD-9-CM Code | Description |
| --- | --- |
| 135 | Sarcoidosis |
| 237.7 | Neurofibromatosis |
| 272.7 | Lipidoses |
| 277.3 | Amyloidosis |
| 277.8 | Other specified disorders of metabolism—includes eosinophilic granuloma |
| 446.21 | Goodpasture’s syndrome |
| 446.4 | Wegener’s granulomatosis |
| 495 | Extrinsic allergic alveolitis |
| 500 | Coal workers’ pneumoconiosis |
| 501 | Asbestosis |
| 502 | Pneumoconiosis due to other silica or silicates |
| 503 | Pneumoconiosis due to other inorganic dust |
| 504 | Pneumoconiosis due to inhalation of other dust |
| 505 | Pneumoconiosis, unspecified |
| 506.4 | Chronic respiratory conditions due to fumes or vapors |
| 508.1 | Chronic and other pulmonary manifestations due to radiation |
| 508.8 | Respiratory conditions due to other specified external agents |
| 515 | Postinflammatory pulmonary fibrosis |
| 516 | Pulmonary alveolar proteinosis |
| 516.1 | Idiopathic pulmonary hemosiderosis |
| 516.2 | Pulmonary alveolar microlithiasis |
| 516.8 | Other specified alveolar and parietoalveolar pneumonopathies |
| 516.9 | Unspecified alveolar and parietoalveolar pneumonopathies |
| 517.2 | Lung involvement in systemic sclerosis |
| 517.8 | Lung involvement in other diseases classified elsewhere |
| 518.3 | Pulmonary eosinophilia |
| 555 | Regional enteritis |
| 710 | Systemic lupus erythematosus |
| 710.1 | Systemic sclerosis |
| 710.2 | Sjo¨ gren’s disease |
| 710.3 | Dermatomyositis |
| 710.4 | Polymyositis |
| 714.81 | Rheumatoid lung |
| 720 | Ankylosing spondylitis |
| 759.5 | Tuberous sclerosis |

Definition of abbreviation: ICD-9-CM International Classification of Diseases, 9th edition, Clinical Modification.

]
