# Supplementary material for: Epidemiologic Analysis of Taiwanese Patients with Idiopathic Pulmonary Fibrosis
Source: Healthcare (Basel). 2020 Dec 21;8(4):580. doi: 10.3390/healthcare8040580 (PMC7767390; doi:10.3390/healthcare8040580)
Supplement: Supplementary file 1 [file healthcare-08-00580-s001.zip › Supplmentary Table S3.docx.doc]

Table S3. ICD-9-CM Codes for Respiratory Causes Hospitalization

| ICD-9-CM Code | Description |
| --- | --- |
| 480.8 | Pneumonia due to other virus not elsewhere classified |
| 481 | Pneumococcal pneumonia |
| 482.41 | Methicillin susceptible pneumonia due to Staphylococcus aureus |
| 482.8 | Pneumonia due to other specified bacteria |
| 484.1 | Pneumonia in cytomegalic inclusion disease |
| 484.3 | Pneumonia in whooping cough |
| 484.5 | Pneumonia in anthrax |
| 484.6 | Pneumonia in aspergillosis |
| 484.7 | Pneumonia in other systemic mycoses |
| 484.8 | Pneumonia in other infectious diseases classified elsewhere |
| 486 | Pneumonia, organism unspecified |
| 487 | Pneumonia with Influenza |
| 494 | Bronchiectasis |
| 491.21 | Obstructive chronic bronchitis with acute exacerbation |
| 518.81 | Acute respiratory failure |
| 518.82 | Other pulmonary insufficiency, not elsewhere classified |
| 518.83 | Chronic respiratory failure |
| 518.84 | Acute and chronic respiratory failure |
| 786.09 | Other dyspnea and respiratory abnormalities |
| 516.3 | Idiopathic fibrosing alveolitis |
| 515 | Postinflammatory pulmonary fibrosis |
| 799.02 | Hypoxemia |

Definition of abbreviation: ICD-9-CM International Classification of Diseases, 9th edition, Clinical Modification.
